# Review of brain-computer interface technology in ophthalmology: Current status, challenges and future directions

**DOI:** 10.1016/j.aopr.2026.03.005

**Published:** 2026-03-25

**Authors:** Jie Zhou, Dongyu Hu, Meichen Wu, Zichao Hong, Rong li, Qianyin Chen, Xuesong Mi, Jinglin Zhang

**Affiliations:** Department of Ophthalmology, The First Affiliated Hospital of Jinan University, Guangzhou, China

**Keywords:** Brain-computer interface (BCI), Visual impairment, Retinal prosthesis, Visual cortical prosthesis, Noninvasive brain stimulation, Glaucoma, Artificial visual restoration

## Abstract

**Background:**

Visual impairment is a major global public health issue. Irreversible blindness caused by end-stage outer retinal diseases, optic nerve injuries, and other conditions remains refractory to conventional therapies. Brain-Computer Interface (BCI) technology, which establishes a direct communication pathway between the brain and external devices, has emerged as a promising interdisciplinary strategy for ophthalmic diagnosis, functional assessment, and artificial visual restoration.

**Main text:**

This review summarizes recent advances in BCI technology for ophthalmic applications. In diagnosis and assessment, BCIs provide objective and quantitative measures for evaluating visual disorders and the integrity of the visual pathway through neural signals, using modalities such as steady-state visual evoked potentials, functional near-infrared spectroscopy, and functional magnetic resonance imaging. In treatment, implantable visual prostheses, particularly retinal and cortical prostheses, have shown substantial progress in partial visual reconstruction, while noninvasive BCI-related approaches are being increasingly explored for rehabilitation. However, major barriers remain, including difficulties in signal acquisition, immature encoding and decoding algorithms, limited electrode array performance, inefficient wireless transmission, implantation-related complications, high device costs, and insufficient evidence for some noninvasive interventions. Legal, regulatory, and ethical concerns also constrain large-scale clinical implementation.

**Conclusions:**

BCI technology holds considerable promise in ophthalmology, but significant technical and translational challenges remain. Future advances in artificial intelligence, flexible electronics, virtual reality, wireless systems, and closed-loop strategies are expected to improve precision, safety, adaptability, and accessibility, thereby enabling more effective diagnostic and visual rehabilitation solutions for patients with severe visual impairment.

## Highlights

• Brain–computer interfaces are a rapidly expanding research focus.
• Ophthalmic applications of BCIs remain insufficiently synthesized.
• This review integrates recent advances across diagnosis and visual prostheses.
• Emerging retinal and visual cortical implants are systematically summarized.
• Current limitations and future clinical prospects are discussed.

## 1 Introduction

Vision serves as the primary channel through which humans acquire external information, forming the cornerstone of human perception, cognition, and behavior.^1^^,^^2^ Accurate visual input relies on the structural and functional integrity of the visual pathway. Irreversible damage to components of this pathway, such as retinal photoreceptors, the optic nerve, and the visual cortex, results in permanent vision loss. For example, patients with retinitis pigmentosa (RP) or age-related macular degeneration (AMD) exhibit progressive degeneration of photoreceptors and retinal pigment epithelium cells. In later stages, while inner retinal cells may remain viable, they lose their source of light-mediated input.^3^^,^^4^ Conversely, individuals suffering from end-stage glaucoma or traumatic optic nerve injury face necrosis of optic nerve fibers, disrupting the transmission of visual signals to the brain.^5^^,^^6^

According to the World Health Organization, there were approximately 43.3 million blind individuals worldwide in 2020, with projections suggesting an increase to 61 million by 2050, creating a significant socioeconomic burden.^7^ Current conventional medical and surgical interventions have shown limited efficacy for advanced cases. In contrast, emerging therapies such as gene therapy, optogenetics, and stem cell-based treatments are still in early clinical trial phases, leaving their therapeutic efficacy unvalidated.^3^^,^^8^, ^9^, ^10^

Brain-Computer Interface (BCI) systems, situated at the intersection of neuroengineering and biomedicine, are crucial for applications ranging from motor function restoration to cognitive training by detecting and decoding diverse neural signals.^11^ Given that the eye is the brain's primary sensory organ for visual information, BCI technology can play a substantial role in ophthalmology.^12^^,^^13^ Specifically, in this context, BCI systems support both functional assessment and artificial visual reconstruction. They facilitate early screening and quantitative evaluations of ophthalmic disorders by monitoring neural responses to visual stimuli. For therapeutic applications, BCIs can restore artificial vision through targeted stimulation along the visual pathway. Since Dobelle's pioneering work eliciting phosphenes in blind subjects through electrical stimulation of the visual cortex,^14^ ophthalmic BCI technology has progressed from preclinical research to clinical translation, suggesting promising applications for acquired blindness therapies.^15^

This review will summarize the technical principles, preclinical results, and clinical findings associated with recent advancements in BCI technology for ophthalmic applications. It will also address key challenges in translating BCI technology into standard clinical practice and propose future research directions aimed at overcoming these barriers, ultimately providing guidance for both basic science and clinical research efforts.

## 2 Diagnosis and assessment

In BCI-based diagnostic and assessment paradigms, neural signals are used to evaluate the integrity of the visual pathway by quantifying responses to visual stimulation. Modalities fall into invasive and noninvasive categories. Invasive methods provide superior spatiotemporal resolution at the cost of surgical risk; noninvasive methods are safer and more scalable but require advances in signal quality and decoding.^16^ Common invasive (intracranial) signals include electrocorticography (ECoG) and single-neuron action potentials (spikes); ECoG is sometimes classified as semi-invasive because electrodes are positioned epidurally or subdurally rather than inserted into cortical tissue.^17^ Noninvasive modalities include electroencephalography (EEG), magnetoencephalography, functional magnetic resonance imaging (fMRI), and functional near-infrared spectroscopy (fNIRS).^16^ Here, we focus on several neural signals modalities that are commonly used in ophthalmic diagnosis and assessment.

### 2.1 Steady-state visual evoked potential (SSVEP)

Visual stimulation elicits periodic EEG responses in visual cortex at the stimulus frequency, producing SSVEPs when frequency is constant.^18^ Traditional SSVEP decoding approaches commonly use canonical correlation analysis and task-related component analysis for feature extraction and target recognition.^19^ In recent years, deep learning models incorporating tensor-based feature fusion^20^ and spatial-frequency dual-branch feature extraction^21^ have further improved the representation and decoding performance of EEG signals. As an objective and sensitive biomarker, SSVEPs have been used for both diagnosis and visual function assessment. For example, Nakanishi et al.^22^ developed a portable BCI device called nGoggle based on multifocal SSVEP (mfSSVEP) for glaucoma screening. Its area under the receiver operating characteristic curve was reported to be 0.924, with better portability and accuracy than standard automated perimetry, along with advantages such as support for automated diagnosis and streamlined computational processes,^23^ enabling earlier detection of the disease. In addition, intermodulation frequencies in the SSVEP spectrum can further assess visual integration deficits (such as cataracts, strabismus, and amblyopia) and quantify treatment responses.^24^ SSVEPs are also well suited to objective assessment of visual function. SSVEP-based visual acuity paradigms can estimate monocular visual acuity in approximately 2 mins. This approach improves testing efficiency and reduces visual fatigue, making it particularly suitable for individuals unable to complete subjective visual acuity tests reliably.^25^ SSVEP has also been applied to the assessment of color vision^26^ and contrast sensitivity.^27^ Overall, SSVEPs offer practical advantages, including high information transfer rates, multiple command options, and minimal training requirements; however, they depend on exogenous visual stimulation and prolonged fixation may induce fatigue. When appropriately deployed, SSVEPs provide robust, objective endpoints for diagnosis and longitudinal monitoring.

### 2.2 Functional near-infrared spectroscopy (fNIRS)

fNIRS is a noninvasive, portable neuroimaging technique that quantifies cortical oxyhemoglobin and deoxyhemoglobin concentration changes via near-infrared light absorption, serving as an indirect surrogate for neural activity.^28^ Given that distinct visual stimuli produce heterogeneous activation responses in the occipital cortex, fNIRS has been proven effective for visual function assessment by detecting stimulus-specific hemodynamic changes in the visual cortex.^29^ In a pilot study, compared with healthy individuals, glaucoma patients exhibited a significant reduction in occipital cortical activity following visual stimulation tasks, and the degree of this reduction was positively correlated with retinal nerve fiber layer thickness and the severity of visual field defects.^30^ Furthermore, fNIRS can be combined with other neural signals, such as EEG, to detect changes in brain networks in real time, which holds great significance for evaluating the efficacy of visual training and investigating the neural mechanisms of the brain.^31^^,^^32^ Advantages include portability, safety, and ecological validity for studies outside the lab. Signal quality, however, varies with hair density/color and skin pigmentation due to light absorption and scattering.^33^ Wider use of high-sensitivity devices, standardized protocols, and diverse cohorts should mitigate these limitations and expand clinical utility.

### 2.3 Functional magnetic resonance imaging (fMRI)

The core principle of fMRI relies on the blood oxygen level-dependent (BOLD) effect: increased neural activity boosts cerebral blood flow far more than oxygen metabolism, reducing deoxyhemoglobin, altering local magnetic field homogeneity, and inducing detectable magnetic resonance signal changes for noninvasive dynamic brain imaging.^34^ fMRI has been widely applied in clinical ophthalmology and basic research. For example, BOLD-based retinotopic mapping can reveal nonresponsive cortical regions corresponding to atrophic retina^35^ and serve as an objective criterion for evaluating the therapeutic efficacy of neovascular AMD at the cortical level.^36^ In addition, reduced cerebral cortical activity directly reflects lesions in the visual pathway and correlates with the degree of visual field defects.^37^ Thanks to its advantages of high spatial resolution and whole-brain coverage, fMRI serves as an excellent tool for analyzing the neural basis of distributed network interactions and complex behaviors, facilitating research on cortical plasticity in diseases such as amblyopia, glaucoma, and AMD.^38^^,^^39^ For instance, fMRI studies have shown that patients with AMD exhibit significant functional brain changes^39^: resting-state fMRI reveals abnormal functional connectivity across multiple brain regions (with enhanced connectivity in frontal and parietal lobes and reduced connectivity in cerebellum, cingulate gyrus, and thalamus), while task-based fMRI demonstrates attenuated activation in visual cortices (e.g., V1) and compensatory activation in non-visual areas (such as prefrontal and parietal cortices), reflecting the brain's adaptive neural plasticity in response to retinal damage. However, fMRI's temporal resolution is limited, and motion and physiological artifacts are common; equipment and operating costs restrict routine clinical use. Future directions include eye-tracking-informed artifact correction, dedicated ophthalmic devices, and multimodal integration with EEG/fNIRS, enabling earlier functional assessment and individualized therapy planning.

### 2.4 Electrocorticography (ECoG)

ECoG records local field potentials directly from the cortical surface, reducing skull/scalp attenuation and improving spatial specificity.^40^ In vision research, broadband gamma (110–140 Hz) can differentiate stimulus attributes such as type and color, suggesting diagnostic potential in color perception disorders.^41^ Direct cortical contact enables precise mapping and electrical stimulation, but invasiveness limits routine use; anesthesia, analgesia, and surgery can also alter signals. Consequently, ECoG currently suits intraoperative mapping, advanced diagnostics, and closed-loop prosthesis development more than standard clinic deployment.

In summary, BCI applications in ophthalmic diagnosis and assessment offer several key advantages. They provide objective, quantitative measures that are well suited to special populations, and they enable multimodal approaches that can address diverse clinical needs, supporting early screening and mechanistic investigations. However, important limitations remain: noninvasive methods are sensitive to signal quality and other confounds; invasive approaches are difficult to scale for routine clinical use; and some systems are costly and vulnerable to interference. Future work should prioritize multimodal integration, device optimization, and methodological standardization to accelerate clinical translation toward earlier, more precise diagnosis and treatment, as well as reliable long-term monitoring.

## 3 Implantable visual prostheses

Implantable visual prostheses represent a major therapeutic direction for ophthalmic BCI technology. By establishing a direct interface between neural tissue and external devices, these systems aim to restore partial visual perception through electrical stimulation at different nodes of the visual pathway (e.g., retina, optic nerve, or visual cortex).^42^ Such prostheses offer a potential option for patients with blinding diseases refractory to conventional treatment, including AMD and RP. Previous reviews have comprehensively summarized the principles and clinical trial outcomes of various visual prostheses.^43^^,^^44^ Accordingly, we focus on recent technological progress and representative contemporary devices (Table 1), while discussing discontinued platforms only when necessary for context.

**Table 1:** Core information summary of BCI visual prosthesis devices.

Device	Developer/Company	Implantation Site	Electrode Specifications	Electrode Material/Coating	Key Advantages	Clinical Trial Number(s)	Study Sample/Follow-up
Argus II	Second Sight Medical Products, Sylmar, California, USA	Epiretinal	60-channel epiretinal surface microelectrode array (6 × 10); electrode diameter: 200 μm; center-to-center spacing: 575 μm	Electrode core: Platinum	Multi-country regulatory approval and broad clinical use; long-term safety and efficacy demonstrated; improved orientation, activities of daily living, and social interaction; evidence of adult visual system neuroplasticity	NCT03635645, NCT02747589	Approximately 400 implanted worldwide,^43^ 30 patients with >5-year follow-up^45^
IMIE 256	Golden Eye Bionic, Pasadena, California, USA; IntelliMicro Medical, Changsha, Hunan, China	Epiretinal	256 electrodes (248 large electrodes, 210 μm diameter; 8 small electrodes, 160 μm diameter); functional yield: 99.9%	Electrode surface coating: Platinum gray	High electrode density for finer visual stimulation; low serious adverse event (SAE) rate; retina-conforming design for easier implantation; significant visual function improvement; flexible external system with dual cameras	None	n = 5; 90-day follow-up^46^
POLYRETINA	Swiss Federal Institute of Technology Lausanne, Lausanne, Switzerland	Epiretinal	Basic version: 2215 stimulation pixels (80/130 μm diameter); high-density version: 10498 independent photovoltaic pixels; pixel density: 79.1 pixels/mm^2^	Electrode surface coating: Ti/TiN	Combined wide visual field and high pixel density; passive wireless design with reduced surgical invasiveness; high biocompatibility and stability; high spatial selectivity with minimal electrical crosstalk	None	Animals: 11 pigs (3 implanted with 2-week follow-up)^47^
PRIMA	Pixium Vision, Paris, France (acquired by Science Corporation, San Francisco, California, USA)	Subretinal	378 photovoltaic pixels; each pixel integrates active and return electrodes	Electrode material: Metal; next-generation version includes titanium sputtering coating	Compatibility with artificial central vision and natural peripheral vision; minimally invasive wireless design; meaningful visual improvement for functional tasks; potential for in situ chip replacement and system upgrading	NCT03333954, NCT04676854	n = 38 implanted; 32 completed 12-month follow-up; 5 completed 4-year follow-up^48^
Suprachoroidal Device	Bionic Vision Australia, Melbourne, Victoria, Australia	Suprachoroidal	44 platinum stimulating electrodes in a staggered grid plus 2 large platinum return electrodes (46 total functional units)	Electrodes core: Platinum	Reduced surgical trauma and favorable safety profile; good long-term stability with 97% electrode function retention; support for multimodal stimulation and algorithm upgrades; wireless and portable home-use capability	NCT01603576, NCT03406416, NCT05158049	Generation 1: n = 3; 12-month follow-up^49^; Generation 2: n = 4; 2-year follow-up^50^
ORION	Cortigent, Salt Lake City, Utah, US	Subdural	60-electrode array	Electrode core: Platinum (clinical version); sputtered platinum gray (research version)	Favorable surgical safety profile; stable percept generation with low localization error; functional adaptability for daily use; excellent long-term reliability	NCT03344848, NCT02747589	n = 6; up to 6-year follow-up^51^
CORTIVIS	Bioengineering Institute, University Miguel Hernández, Elche, Valenciana Community, Spain	Intracortical	96- or 100-channel Utah Electrode Array; electrode length: 1.5 mm; spacing: 400 μm	Electrode core: Platinum-iridium alloy	High spatial resolution with 400 μm two-point discrimination; good short-term functional stability; combined stimulation and recording capability; minimally invasive craniotomy with rapid recovery	NCT02983370	n = 4; 6-month follow-up^52^^,^^53^^,^^54^
Gennaris	Monash Vision Group (MVG), Monash University, Clayton, Victoria, Australia	Intracortical	43 penetrating intracortical microelectrodes per array; up to 11 arrays (473 electrodes total) plus an annular return electrode	Electrode core: Platinum-iridium alloy	Highly integrated wireless architecture with reduced infection risk; precise stimulation with low power consumption; favorable surgical adaptability; modular design allowing flexible system configuration	None	Animal study: 3 sheep; follow-up 164–276 days^55^
ICVP	Illinois Institute of Technology (IIT), Chicago, Illinois, USA	Intracortical	16 stimulation electrodes plus 1 reference electrode per wireless floating microelectrode array (WFMA); Subject 1: 25 WFMAs (400 electrodes); Subject 2: 32 WFMAs (512 electrodes)	Electrode core: Platinum-iridium alloy	Wireless and highly integrated design for broad cortical coverage; precise stimulation with safety monitoring, including EEG-based seizure warning; good long-term clinical stability	NCT04634383	n = 2; 1 patient with 3-year follow-up^56^

### 3.1 Retinal prostheses

**Fig. 1:**
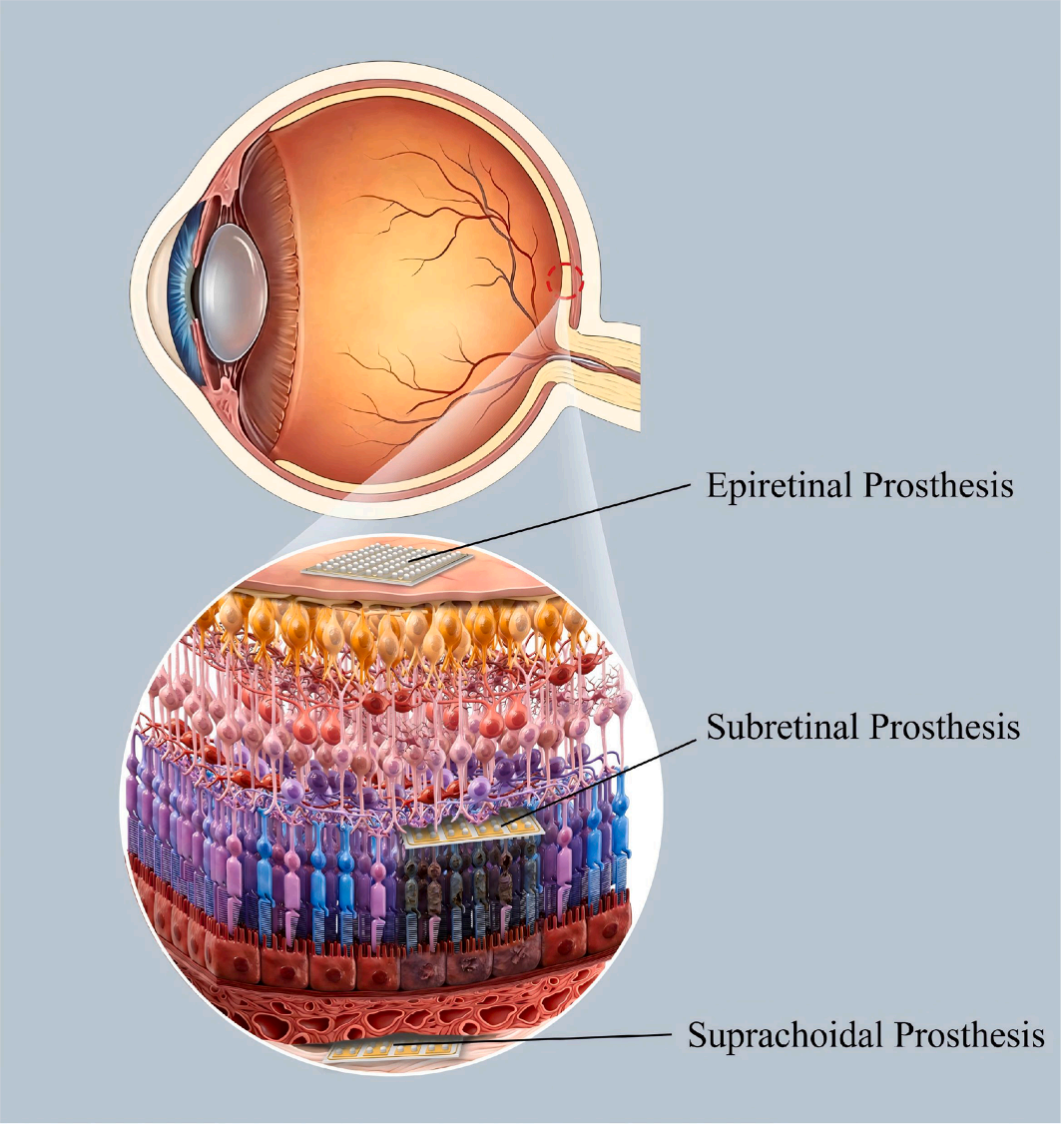
**Schematic illustration of retinal prosthesis implantation sites.** Retinal prostheses may be classified according to the location of the implanted electrode array as epiretinal, subretinal, or suprachoroidal prostheses.

Retinal prostheses can be categorized according to the anatomical location of the implanted electrode array, including epiretinal, subretinal, and suprachoroidal prostheses. As illustrated in Fig. 1, epiretinal devices are placed on the inner retinal surface, subretinal devices are positioned beneath the neural retina, and suprachoroidal devices are implanted between the choroid and sclera.

#### 3.1.1 Epiretinal prostheses

Epiretinal prostheses place an electrode array on the inner retinal surface to stimulate retinal ganglion cells directly. The most widely recognized example is the Argus II Retinal Prosthesis System (Second Sight Medical Products), which previously obtained CE marking and U.S. Food and Drug Administration (FDA) approval and was implanted in approximately 400 patients worldwide for end-stage outer retinal degeneration. The system captures images via a camera mounted on glasses, processes and encodes signals in an external unit, transmits data wirelessly to an implanted receiver coil, and delivers stimulation through a 60-channel epiretinal electrode array.^57^ Clinical reports indicate improvements in functional tasks such as localization and mobility, with benefits persisting for up to 5 years in some cohorts.^45^, ^58^, ^59^ Neuroimaging studies further suggest enhanced responsiveness of the primary visual cortex and lateral geniculate nucleus with long-term use, consistent with neuroplastic adaptation.^60^^,^^61^ However, the clinical application of the Argus II system is subject to several notable limitations. In one long-term study, all 10 patients in that cohort had discontinued use of the device (mean usage: 3.34 years) and reported a modest satisfaction rating, primarily due to poor performance, suboptimal visual quality, and substantial cognitive burden.^62^ Additionally, implantation of the system is associated with a range of complications, including conjunctival erosion, retinal fibrosis, retinal detachment, device malfunction, and device displacement.^45^^,^^59^^,^^63^, ^64^, ^65^ From a mechanistic perspective, retinal fibrosis may be induced by several factors, including mechanical contact between the electrode array and the retina, abnormal proliferation of residual vitreous tissue triggered by electrical stimulation, and retinal injury resulting from the combined effects of mechanical pressure and electrical stimulation. Such fibrosis can subsequently lead to secondary pathological changes, including retinal traction and even retinal detachment. These challenges place stringent demands on the biocompatibility and mechanical compliance of materials used in visual prostheses. The platform was discontinued in 2019 because of financial constraints, raising concerns regarding long-term device support and management of late complications.^64^ Despite these limitations, Argus II remains a milestone that catalyzed retinal prosthesis development and interdisciplinary translation.

**Fig. 2:**
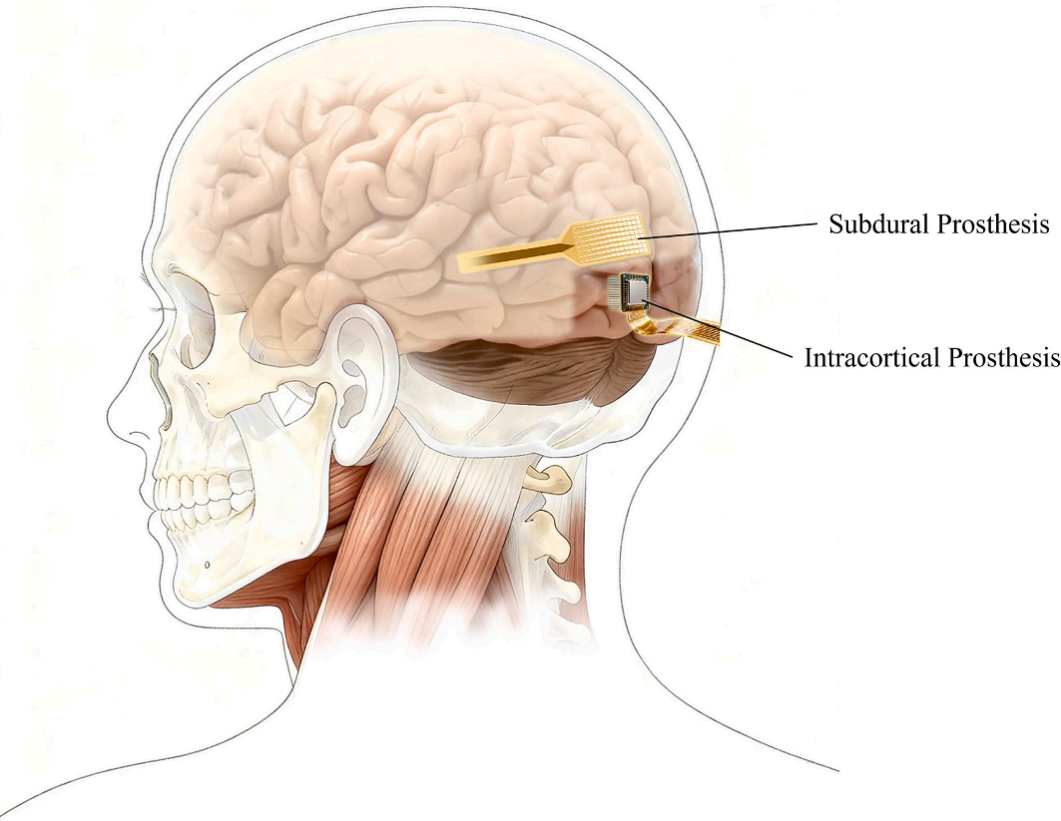
**Schematic illustration of the anatomical implantation sites of cortical visual prostheses.** Cortical visual prostheses can be classified according to the location of the implanted electrode array as subdural or intracortical prostheses.

Building on Argus II, newer epiretinal systems have pursued higher electrode counts (e.g., IMIE256, NR600) to improve spatial resolution,^46^^,^^66^ optimized array designs to reduce stimulation thresholds and adverse events,^66^ improved curvature and conformability to minimize retinal trauma and expand visual field^46^^,^^47^, ^67^, ^68^, and adopted advanced biomaterials and flexible electrodes to enhance biocompatibility and reduce mechanical injury.^46^^,^^69^ The POLYRETINA system, for instance, uses a foldable implant designed for small-incision insertion.^47^ An alternative minimally invasive strategy has also been explored using intravitreally injected, targeted near-infrared–absorbing plasmonic gold nanorods to preferentially activate inner retinal neurons and elicit cortical responses in animal models without overt systemic toxicity.^70^ Although most current epiretinal prosthesis clinical trials have not achieved ideal outcomes, and some devices have been discontinued due to technical bottlenecks or funding issues, the continuous emergence of new materials and schemes is steadily advancing the field of artificial vision toward greater safety and efficacy (Fig. 2).

#### 3.1.2 Subretinal prostheses

Subretinal prostheses are implanted beneath the retina to replace degenerated photoreceptors and primarily stimulate bipolar cells, allowing downstream signal processing through remaining retinal circuitry before transmission to the brain via retinal ganglion cells. A representative device is the PRIMA (Photovoltaic Retina Implant Microarray) system (Pixium Vision; now acquired by Science Corporation), which includes 378 hexagonal pixels (100 μm) that convert projected pulsed near-infrared (880 nm) light into local electrical currents, selected to minimize photophobia and phototoxicity.^71^ Follow-up studies on patients with geographic atrophy have demonstrated that the PRIMA system enables the simultaneous perception of central vision and natural peripheral vision while maintaining stable safety profiles.^72^ Over a 4-year follow-up period of 3 patients, their visual acuity closely aligned with the pixel size of the implant, reaching a mean of 1.39 logMAR.^73^ When the magnification function was utilized, visual acuity improved by 32 ETDRS letters compared to baseline, with no adverse impacts on peripheral vision. In a larger-scale multicenter study involving 38 patients followed for 12 months, 81% of the participants achieved a clinically meaningful improvement in visual acuity (≥0.2 logMAR), with an average improvement of 0.51 logMAR (equivalent to 25.5 letters).^48^ In addition, 84% of participants were able to read letters, numbers, or words at home using the PRIMA system. With the aid of digital functions integrated into the accompanying glasses, including zoom (1–12 × magnification) and contrast enhancement, patients were able to perform daily visual tasks such as reading and writing. The research team has upgraded the implant, reducing the pixel size from 100 μm to 22 μm, which is expected to improve visual acuity from 20/420 to 20/80. The optimized electrode structure offers higher stimulation efficiency and better adaptation to the retina, while enabling reversible explantation of the device.^74^ Moreover, emerging materials are enabling subretinal prostheses to move beyond conventional vision restoration. For example, an ultra-broad-spectrum subretinal nanoprothesis based on tellurium nanowire networks restored visible-light vision in blind animal models and also enabled detection of infrared light beyond the normal visual range.^75^ However, subretinal implantation remains invasive and carries risks such as elevated intraocular pressure and retinal detachment. Functional outputs are currently limited (e.g., monochromatic vision), and quality-of-life gains may remain insufficient for many daily tasks. Continued progress is expected through material innovation, surgical refinement (including minimally invasive and reversible implantation), and adaptive stimulation strategies supported by artificial intelligence (AI).

#### 3.1.3 Suprachoroidal prostheses

Suprachoroidal prostheses position electrodes between the choroid and sclera, stimulating outer retinal tissues while avoiding vitreoretinal surgery. Bionic Vision Australia has advanced this approach through iterative clinical development. The first-in-human trial of a 33-electrode device demonstrated feasibility and early functional benefit (e.g., light localization) in patients with advanced RP.^49^ A second-generation 44-electrode system adopted a fully implantable design to reduce infection risk and improve usability. Clinical and real-world studies reported favorable safety (no serious adverse events, with only minor electrode function loss) and improvements in localization, motion discrimination, and obstacle avoidance, supporting mobility and independence. Long-term follow-up (about 2.7 years) suggested sustained implant stability after early mild displacement.^76^^,^^77^ Algorithmic advances (e.g., face and object detection pipelines) further improved performance in socially relevant tasks.^50^, ^78^, ^79^ Limitations include higher stimulation thresholds due to greater distance from the retina and an electrode array that does not naturally track eye movements. Further optimization and expanded clinical validation are required to define long-term benefits and ideal candidate populations.

### 3.2 Visual cortical prostheses

Retinal prostheses require preserved inner retina and an intact optic nerve. In contrast, cortical prostheses can bypass damaged anterior visual pathway structures if the visual cortex remains functional. Moreover, cortical surface area supports potentially broader visual field representation than the limited intraretinal space, making cortical visual prostheses a major focus of recent research. Cortical visual prostheses may be categorized as subdural or intracortical systems according to the location of the implanted electrodes. As illustrated in Fig. 2, subdural electrode arrays are positioned on the cortical surface, whereas intracortical microelectrode arrays penetrate the cortex and directly stimulate neurons within the visual cortex.

#### 3.2.1 Orion

The Orion visual cortical prosthesis (Cortigent, Salt Lake City, Utah, USA) is a subdural cortical implant comprising a 60-electrode array (2 mm in diameter) and an implanted intracranial processing and control module. During the early research and development phase, the investigators implanted the FDA-approved NeuroPace Responsive Neurostimulation System, originally developed for epilepsy treatment, into the right occipital cortex of a patient with Vogt–Koyanagi–Harada disease who had been blind for 8 years. Over a 19-month observation period, phosphenes elicited by stimulation of each electrode contact were highly consistent within and across experimental sessions, exhibiting only minor variability in position and size as well as comparable temporal fluctuations. This seminal work established the feasibility, safety, and perceptual stability required for subsequent clinical trials of the Orion visual cortical prosthesis.^80^ In addition, the research team found that the number of perceived phosphenes was strongly correlated with inter-electrode spacing. Simultaneous stimulation of electrode pairs separated by more than 4 mm typically evoked two distinct phosphene percepts, whereas stimulation of three or more electrodes often generated stable spatial phosphene patterns.^81^ However, the absolute position, size, and orientation of these patterns varied considerably across trials. In subsequent studies specifically involving Orion implant recipients, testing in two participants with only residual light perception showed that simultaneous stimulation of a sufficient number of electrodes under static stimulation paradigms was still unable to produce coherent shape perception. By contrast, a temporally sequenced stimulation strategy combined with current steering enabled blind participants to accurately identify seven letter shapes using dynamic stimulation delivered through only five physical electrodes.^82^ Furthermore, a combined strategy involving bimanual fixation and logarithmic model fitting achieved highly accurate and stable phosphene localization, significantly improving mapping reliability in blind participants.^83^ These findings suggest that physical resolution and perceptual resolution are not simply linearly related, but instead depend largely on how efficiently the physical electrodes are utilized through specific stimulation paradigms and strategies. Collectively, these findings suggest that visual cortical prostheses are capable of supporting not only isolated visual percepts but also structured, higher-order perceptual representations. Such observations provide important insights for the future development of visual cortical prostheses and other BCI applications, while also advancing our understanding of the relationship between patterned cortical stimulation and visual perception. According to a 2026 report by Cortigent,^51^ a 6-year early feasibility study of the ORION system in six blind participants suggested that implantation may improve functional vision, including object identification and motion detection. Electrode functionality remained above 96%, and one early-onset seizure was reported as a serious adverse event. A large-scale pivotal clinical trial is planned to further assess the system for potential marketing approval.

#### 3.2.2 Implanted Cortical Visual Prosthesis (ICVP)

The ICVP is a visual cortical prosthesis composed of multiple Wireless Floating Microelectrode Arrays (WFMAs), each containing 16 stimulating electrodes. In an FDA-authorized phase I clinical trial (NCT04634383), 25 WFMAs were implanted into the right occipital visual cortex of a participant with only light perception. At 9-month follow-up, electrode performance remained stable, and the participant achieved a grating visual acuity of 2.3 logMAR or better, representing the first reported functional visual outcome from an ICVP in a human.^84^ When integrated with a visible-light camera or a thermal camera, the ICVP was used to provide real-time visual cues that supported walking direction discrimination and identification of occupied versus vacant chairs in proof-of-concept testing.^85^^,^^86^ Safety considerations remain critical: interviews with family members of 12 of 16 individuals who had received visual cortical implants revealed that three experienced generalized epileptic seizures, which were attributed to cortical stimulation. Consistent with these observations, concurrent scalp EEG monitoring has been shown to enable real-time detection of stimulation-induced epileptiform activity and may provide early warning during higher-risk stimulation conditions.^56^ Future studies aim to enroll additional participants to further evaluate visual performance under expanded stimulation strategies and to explore the limits of visual function achievable with intracortical stimulation.

#### 3.2.3 CORTIVIS

The CORTIVIS system uses a 96-channel Utah Electrode Array (UEA) with 1.5-mm penetrating electrodes implanted in the patient's right occipital cortex near the occipital pole, adjacent to the V1-V2 boundary. As penetrating microelectrodes, UEAs require lower stimulation currents than surface (epicortical) electrodes and can be placed at higher density, supporting finer spatial resolution. In an exploratory, first-in-human study, a 57-year-old woman with complete blindness from toxic optic neuropathy (16 years) was implanted and followed for 6 months. Electrical stimulation evoked phosphenes on 88 of 96 electrodes; the mean single-electrode threshold was 66.8 ± 36.5 μA, within accepted safety limits. Electrodes spaced as little as 400 μm produced separate percepts, indicating high spatial separability. After training, the participant recognized letters (e.g., I, L, C, V, O) and achieved 100% accuracy in object-localization tasks.^52^ Longitudinal recordings showed stable low-frequency signal metrics, with a gradual decline in high-frequency power and interelectrode synchrony (correlation, coherence, phase coherence), a pattern consistent with adaptive cortical remodeling rather than device failure.^87^ The array was explanted at 6 months without complications (no infection, hemorrhage, or cortical injury), supporting procedural safety and reversibility. In a separate case, implantation in a patient without eyes, combined with gaze-tracking, enabled accurate object-location performance, extending applicability to a broader candidate population.^53^ Furthermore, the Cortivis team implanted 100-electrode Utah Arrays in two totally blind participants and conducted simultaneous stimulation and recording over 6 months.^54^ The study showed that neural activity in the visual cortex could accurately predict phosphene perception threshold, brightness, and the minimum discrimination interval, providing important evidence for the development of bidirectional, precision cortical visual prostheses. Overall, the Cortivis studies demonstrated the feasibility, safety, and reversibility of intracortical visual prostheses. These systems not only enabled the elicitation of functional visual percepts, but also achieved a technological breakthrough in bidirectional precise modulation, further broadening their potential applicability and practical value. However, larger-scale and longer-term studies are still needed to confirm their durability and generalizability.

#### 3.2.4 Gennaris

Gennaris is a wireless intracortical visual prosthesis system designed for long-term implantation and developed by Monash University in Australia, consisting of two core components: external devices and internal implanted components. The external components include a head-mounted device with a microcamera, a visual processing unit, and a wireless transmitter and control module, which are responsible for acquiring external visual information, converting it into brain-recognizable stimulation signals, and transmitting them to the internal components via a wireless link. The internal components are fully implantable, encapsulated in biocompatible materials, and tasked with receiving external signals and delivering precise electrical stimulation to the visual cortex. Its core structure includes "Tile devices" and auxiliary fixation structures. Each Tile contains 43 electrodes, and a single system can support 11 Tiles, totaling 473 electrodes.^88^ Ten arrays (7 active arrays and 3 passive arrays) were implanted into 3 sheep, and stimulation was performed through 7 of these devices for 3 months, with a cumulative stimulation time exceeding 2700 h and no adverse health effects observed.^55^ Gennaris is currently entering the human experimental phase.

#### 3.2.5 Neuralink

Neuralink, a prominent BCI company, is developing intracortical interfaces that pair flexible microelectrodes with robotic implantation. An early system consists of 96 ultrathin, flexible threads, each bearing 32 electrodes (3072 in total). A dedicated surgical robot enables micrometer–scale placement to target specific cortical regions while avoiding visible vasculature.^89^ Among its investigational devices, Blindsight—a cortical visual prosthesis—has received FDA Breakthrough Device designation. Blindsight uses a high–channel–count architecture where camera–derived images are encoded into electrical stimulation patterns delivered to the visual cortex to evoke visual percepts in the blind.^90^ In June 2025, Neuralink engineer Joseph O'Doherty reported positive nonhuman–primate results: direct stimulation of vision–related cortex elicited gaze shifts toward virtual targets in more than two–thirds of trials, consistent with induced percepts.^91^ The Blindsight project may begin enrolling patients as early as 2026. Widespread media attention to companies such as Neuralink has further intensified ethical debates surrounding BCIs. Central concerns include the privacy and security of directly collected brain data, the possibility that such technologies may undermine individuals' autonomous decision-making, and the surgical risks of invasive implants coupled with uncertainty regarding their actual benefits.^92^

The encoding and processing of visual information by cortical prostheses involve complex neural activity across multiple brain regions, with marked individual variations among humans. Additionally, the majority of the primary visual cortex is located within the calcarine sulcus, making the selection of stimulation sites and the adjustment of stimulation parameters highly challenging. Meanwhile, visual neural signals themselves are faint and extremely susceptible to noise interference, which poses considerable hurdles for signal decoding. Nevertheless, with the advancement of computer technology, these limitations are being gradually overcome. For example, a study^93^ collected noninvasive scalp EEG signals elicited by visual stimuli. Following basic preprocessing, the radial basis function support vector machine machine learning algorithm effectively decoded stimulus features (e.g., position and size), while the multi-trial probability aggregation strategy significantly improved decoding accuracy. This provides experimental support for the noninvasive optimization of electrical stimulation parameters in visual neuroprostheses.

## 4 Noninvasive BCIs

Noninvasive BCIs are generally safer and more portable than invasive systems, with clinical applications concentrated in two areas: assistive substitution and neurorehabilitation.^94^ In assistive BCIs, external devices are controlled by bypassing damaged neural pathways, thereby restoring lost functions in patients with severe motor impairments. Representative examples include spoken communication substitution for patients with aphasia^95^ and video game control for paralyzed individuals.^96^ However, most current applications rely heavily on visually evoked EEG signals. With advances in BCIs based on alternative sensory modalities, such as audition^97^ and somatosensation,^98^ patients with severe visual impairment may also benefit from advances in BCI technology. In rehabilitative BCIs, neural plasticity is harnessed to promote the reorganization of impaired neural circuits and thereby facilitate the recovery of partially lost functions. For example, noninvasive BCI-assisted training has been shown to significantly improve upper-extremity motor function in post-stroke patients,^99^ and BCIs have also enabled the decoding of urination and defecation motor intentions in patients with spinal cord injury.^100^

Notably, the combination of noninvasive BCIs with noninvasive brain stimulation (NIBS) has emerged as a major research hotspot in recent years, demonstrating considerable translational potential across multiple disorders. When integrated with NIBS techniques such as transcranial magnetic stimulation (TMS), transcranial direct current stimulation (tDCS), and transcranial alternating current stimulation (tACS), BCIs may synergistically enhance neural plasticity through bidirectional modulation, brain-state-dependent precision tuning, and closed-loop feedback along the cortex–spinal cord–peripheral nerve axis.^101^ For instance, Zou et al.^102^ developed a noninvasive closed-loop acoustic BCI for seizure control. This system detects seizure onset by real-time decoding of hippocampal EEG signals using a multilevel threshold model and subsequently triggers targeted ultrasonic vagus nerve stimulation. It demonstrated high biosafety, no obvious thermal effects, and superior acute antiepileptic efficacy compared with conventional electrical vagus nerve stimulation, thereby providing a novel therapeutic strategy and theoretical foundation for noninvasive epilepsy treatment.

NIBS has also attracted growing interest in ophthalmology. In human studies, TMS treatment in adults with amblyopia has been associated with significant improvements in visual acuity, interocular imbalance, and stereopsis.^103^^,^^104^ A review indicated tACS has shown potential benefits in patients with glaucoma and optic neuropathy, whereas tDCS has been more commonly applied in amblyopia, hemianopia, and myopia.^105^ However, another meta-analysis showed that tACS also holds promising application prospects in patients with homonymous hemianopia, as it can significantly improve their visual function^106^ and enhance the effectiveness of perceptual training.^107^ Nevertheless, due to heterogeneous intervention protocols, inconsistent outcome measures, and methodological limitations, robust evidence for the efficacy of NIBS in ophthalmology remains insufficient. Moreover, NIBS-based interventions in ophthalmology have not yet achieved true closed-loop integration with electroencephalographic or other neural signals.

Future studies should emphasize greater methodological rigor, optimization of stimulation parameters, and the integration of real-time neural signals decoding with feedback control, so as to translate these approaches into fully functional BCI systems.^108^ At the same time, personalized therapeutic strategies based on neuroimaging and individual patient characteristics, multimodal integration, closed-loop designs for greater precision, mechanistic investigations, and the expansion of clinical indications will collectively advance noninvasive BCIs toward becoming safer and more effective therapeutic options for visual impairments.^109^

## 5 Conclusions

In summary, BCI technology can support retinal and cortical prosthetic strategies as well as noninvasive approaches in ophthalmology, providing options for functional reconstruction and visual rehabilitation in patients with advanced AMD, RP, and other severe visual disorders. It may also improve ophthalmic diagnostic techniques through the assessment of neural signals. However, substantial challenges remain in clinical translation and practical application. Functionally, current BCI systems struggle to replicate the high resolution, depth perception, and color recognition capabilities of natural vision, and therefore cannot fully meet the demands of daily visual function in patients with visual impairment. Technically, the field is limited by difficulties in signal acquisition, underdeveloped signal encoding algorithms, inadequate electrode array performance, and inefficient wireless transmission. In addition, practical barriers persist, including the high cost of devices and insufficient evidence supporting the efficacy of some noninvasive BCI-related interventions. Large-scale clinical implementation is further constrained by important legal, regulatory, and ethical considerations, such as the risk–benefit balance of invasive procedures and the protection of neural data privacy. Nevertheless, this field still holds immense promise. In the future, through deeper integration with technologies such as AI, flexible electronics, and virtual reality (VR), visual restoration is expected to advance from basic light perception to high-definition shape recognition. Meanwhile, with continued algorithm optimization, the transition to wireless systems, and closed-loop strategies, existing challenges such as signal instability and individual adaptability will be gradually addressed, bringing safer, more efficient, and accessible visual rehabilitation solutions to visually impaired populations worldwide.

## Study approval

Not Applicable.

## Author contributions

The authors confirm contribution to the paper as follows: Conception and design of the review: JZ, XM, JZ; Drafting the manuscript: JZ, DH, MW, ZH, RL, QC; Literature synthesis and organization: JZ, DH, MW, ZH, RL, QC; Revision of the manuscript: QC, XM, JZ. All authors reviewed the results and approved the final version of the manuscript.

## Declaration of generative AI use

During the preparation of this work, the authors used ChatGPT (OpenAI; GPT-5 series) solely to improve language, grammar, and readability. After using this tool, the authors reviewed and edited the content as needed and take full responsibility for the content of the published article.

## Funding

This work was supported by 10.13039/501100010256Guangzhou Science and Technology Plan Project, 2025A03J3392; 10.13039/501100004735Natural Science Foundation of Hunan Province, China, 2023JJ70049.

## Declaration of competing interest

The authors declare that they have no known competing financial interests or personal relationships that could have appeared to influence the work reported in this paper.

## Abbreviations

BCI: Brain-computer interface
AMD: Age-related macular degeneration
RP: Retinitis pigmentosa
SSVEP: Steady-state visual evoked potential
mfSSVEP: Multifocal steady-state visual evoked potential
fNIRS: Functional near-infrared spectroscopy
fMRI: Functional magnetic resonance imaging
BOLD: Blood oxygenation level-dependent
ECoG: Electrocorticography
FDA: Food and Drug Administration
ETDRS: Early Treatment Diabetic Retinopathy Study
ICVP: Implanted Cortical Visual Prosthesis
WFMA: Wireless floating microelectrode array
UEA: Utah Electrode Array
NIBS: Noninvasive brain stimulation
TMS: Transcranial magnetic stimulation
tDCS: Transcranial direct current stimulation
tACS: Transcranial alternating current stimulation
AI: Artificial intelligence
VR: Virtual reality
